# Pairing Colicins B and E5 with Bdellovibrio bacteriovorus To Eradicate Carbapenem- and Colistin-Resistant Strains of Escherichia coli

**DOI:** 10.1128/spectrum.00173-23

**Published:** 2023-04-10

**Authors:** Sumudu Upatissa, Wonsik Mun, Robert J. Mitchell

**Affiliations:** a School of Biological Sciences, Ulsan National Institute of Science and Technology (UNIST), Ulsan, South Korea; Emory University

**Keywords:** antibiotic resistance, *Bdellovibrio*, carbapenems, colicin, colistin, pathogens

## Abstract

While diverse antibacterials are available in nature, each possesses their own strengths and limitations. One such antibacterial is colicins, proteinaceous toxins that are produced by strains of E. coli to subvert the growth or viability of other E. coli strains. Similarly, predatory bacteria, of which Bdellovibrio bacteriovorus is well-known, are microbes that actively predate on and consume other Gram-negative bacterial strains. While they are all quite active as antibacterials, they also present some limitations: rapid resistance development to colicins while predation does not completely kill their prey. Within this study, therefore, we evaluated the impact of two different colicins (colicin B [ColB] and colicin E5 [ColE5]) and B. bacteriovorus HD100 either individually or together against four clinical isolates of E. coli that are resistant to either colistin or carbapenem. While the ColB and ColE5 were quickly active when used alone, causing a significant loss in viability (>3-log) in susceptible populations after only 3 h, the pathogens always grew afterwards and had final cell densities that were similar with their respective controls. Predation with B. bacteriovorus HD100, in contrast, was most pronounced after 24 h (>3-log reduction in each pathogen viability but never complete). When combined, better killing efficiencies were observed with several of the pathogens, with complete eradication realized for two (<100 viable pathogens per mL). Given the diversity of colicins in nature and the broad-spectrum activities of B. bacteriovorus strains, the results presented here suggest there is a massive potential to control pathogens when they are used together.

**IMPORTANCE** The coupled impact of drug resistance with reduced antibiotic development has placed humankind at a postantibiotic crossroads where antibiotic alternatives are desperately needed. Consequently, we discuss here the combined effectiveness of two vastly different classes of antibacterials, namely, colicins and a predatory bacterium (i.e.,Bdellovibrio bacteriovorus HD100), against two priority pathogenic groups, colistin- and carbapenem-resistant strains of E. coli. While each is effective in its own manner, these antibacterials also display limitations, i.e., the rapid appearance of mutations that confer resistance to the colicins while predatory bacteria do not completely kill their prey. Here, we show these limitations can be overcome using combined treatments of these antibacterials, with two pathogenic E. coli populations completely eradicated within 24 h. Given the diversity of colicins and the broad-spectrum activities of B. bacteriovorus strains, the results presented here suggests there is a massive potential to control pathogens when they are used together.

## OBSERVATION

Colicins are proteinaceous toxins produced by some strains of E. coli to kill or thwart the growth of other E. coli strains (1). While colicins have been used in studies to control pathogenic E. coli strains (2, 3), mutations rapidly arise that provide downstream resistance (4), which acts to limit their application. To subvert this, researchers have employed combinatorial approaches where colicins were used alongside other antibacterials, such as bacteriophage or antibiotics (5, 6). A separate class of antibacterials are the *Bdellovibrio*-and-like organisms (BALOs), a group of microbes that actively predate on other Gram-negative bacteria (7, 8). These predators and, in particular, Bdellovibrio bacteriovorus, have received attention recently as a potential alternative to antibiotics as they are active against a wide array of human pathogens (9, 10), killing them while also significantly reducing their antibiotic resistance gene pools (11, 12). BALOs, however, have their own limitations as they are incapable of attacking Gram-positive bacteria (8) or completely killing Gram-negative prey, a phenomenon that has been referred to as “plastic phenotypic resistance” of the prey (13). In contrast with colicins, this “resistant phenotype” is not genetic in nature but rather results from low probabilities for the predator and prey to encounter one another when the latter population drops below a certain threshold density (14). While a couple of studies also explored the use of BALOs with antibiotics or bacteriophage (15, 16), no study has considered the use of BALOs and colicins together.

**FIG 1:**
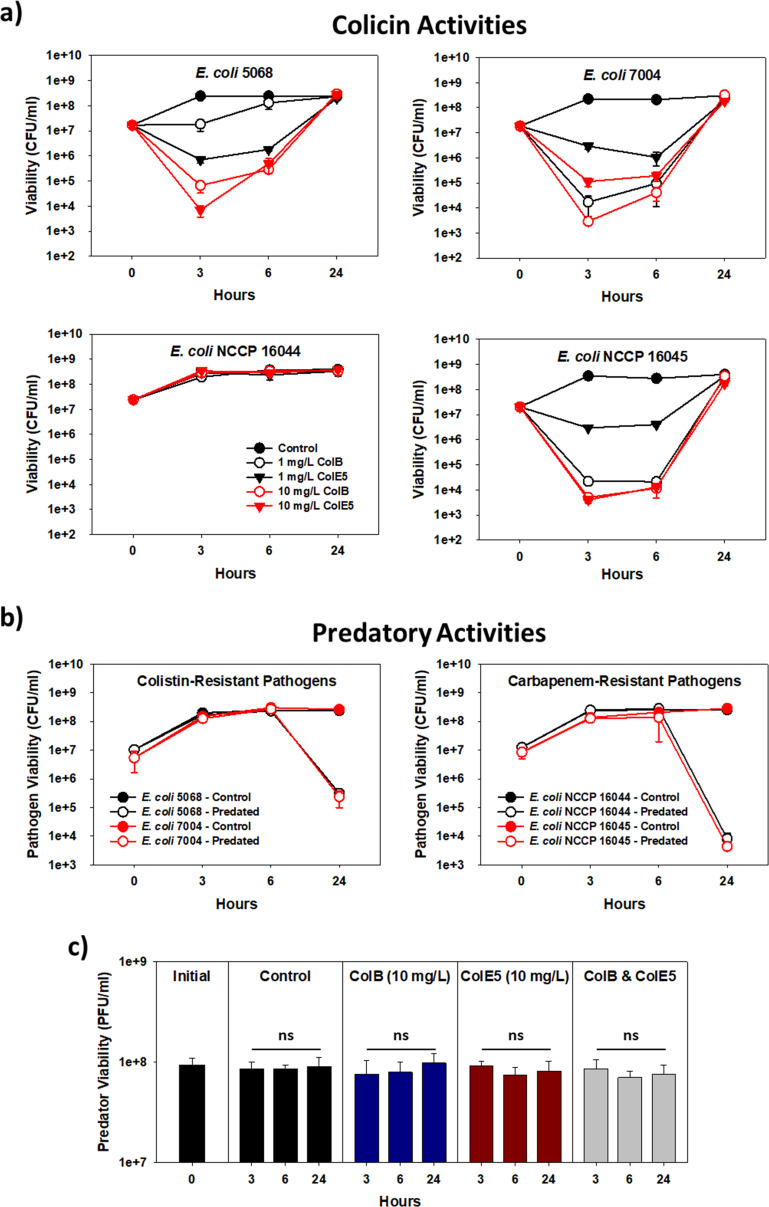
Antibacterial activities of the individual colicins and B. bacteriovorus HD100 in dilute nutrient broth (DNB) medium. (a) ColB and ColE5 activities against the four clinical E. coli isolates in dilute nutrient broth (DNB) medium. The results show three of the pathogens were sensitive to both these proteins, while E. coli NCCP 16044 was inherently resistant to both. The results also illustrate the temporal impact of both colicins as all three sensitive pathogens grew again by 24 h. Based on the results obtained, 10 mg/L was chosen for all subsequent experiments. (*n *= 4). (b) Predation of the four clinical E. coli isolates by B. bacteriovorus HD100 in DNB. The results show all four pathogens were susceptible to predation but, due to a low predator-to-prey ratio (0.02), predation required more than 6 h for any obvious killing to be evident. (*n* = 4). (c) B. bacteriovorus HD100 is not sensitive to ColB or ColE5 in DNB. Neither colicin nor a mixture of the two significantly impacted the predator's viability over 24 h. The Student's *t* test was used to evaluate statistical significance between the different time points. ns – not significant. (*n *= 4).

To evaluate this, therefore, two colicins, colicin B (ColB) and colicin E5 (ColE5), were selected as each present quite different antibacterial activities. ColB is a Group B pore-forming protein that kills the susceptible bacterium by dissipating its proton motive force (17) while ColE5 possesses tRNase activities, causing a halt in protein translation once it enters a susceptible cell (18). The activities of each purified colicin (Fig. S1) against four clinical isolates of E. coli that are either colistin- or carbapenem-resistant (Table S1) are shown in Fig. 1a. Of these pathogens, three were susceptible to both colicins, their cultures experiencing as much as a 5-log loss in viability during the first 6 h, while one (E. coli NCCP 16044) was inherently resistant to both ColB and ColE5. These results were further verified using spot titer assays (Fig. S2). The effectiveness of either colicin against the susceptible strains, however, was clearly short-lived (6 h) as each eventually grew and achieved 24-h viabilities that were akin to those of the untreated controls. Moreover, tests with the surviving E. coli NCCP 16045 cultures found they were now resistant to both colicins (Fig. S3).

Figure 1b shows two benefits of predation over the colicins, namely, all four E. coli were susceptible to predation (including E. coli NCCP 16044), each experiencing a 3- to 5-log loss in viability by 24 h, while regrowth of the E. coli strains was prevented. However, predation was incomplete. Another limitation was the slow initial activity of this predator, which was due to the low initial predator-to-prey ratio (PPR) used (PPR = 0.2). As the colicins acted swiftly (Fig. 1a), we were curious if these two antibacterial classes complement each other when combined. Before we could evaluate this, however, the potential impacts of ColB and ColE5 on the predator needed to be explored. As shown in Fig. 1c, this was not a concern as neither colicin was active against this predator, even when mixed.

**FIG 2:**
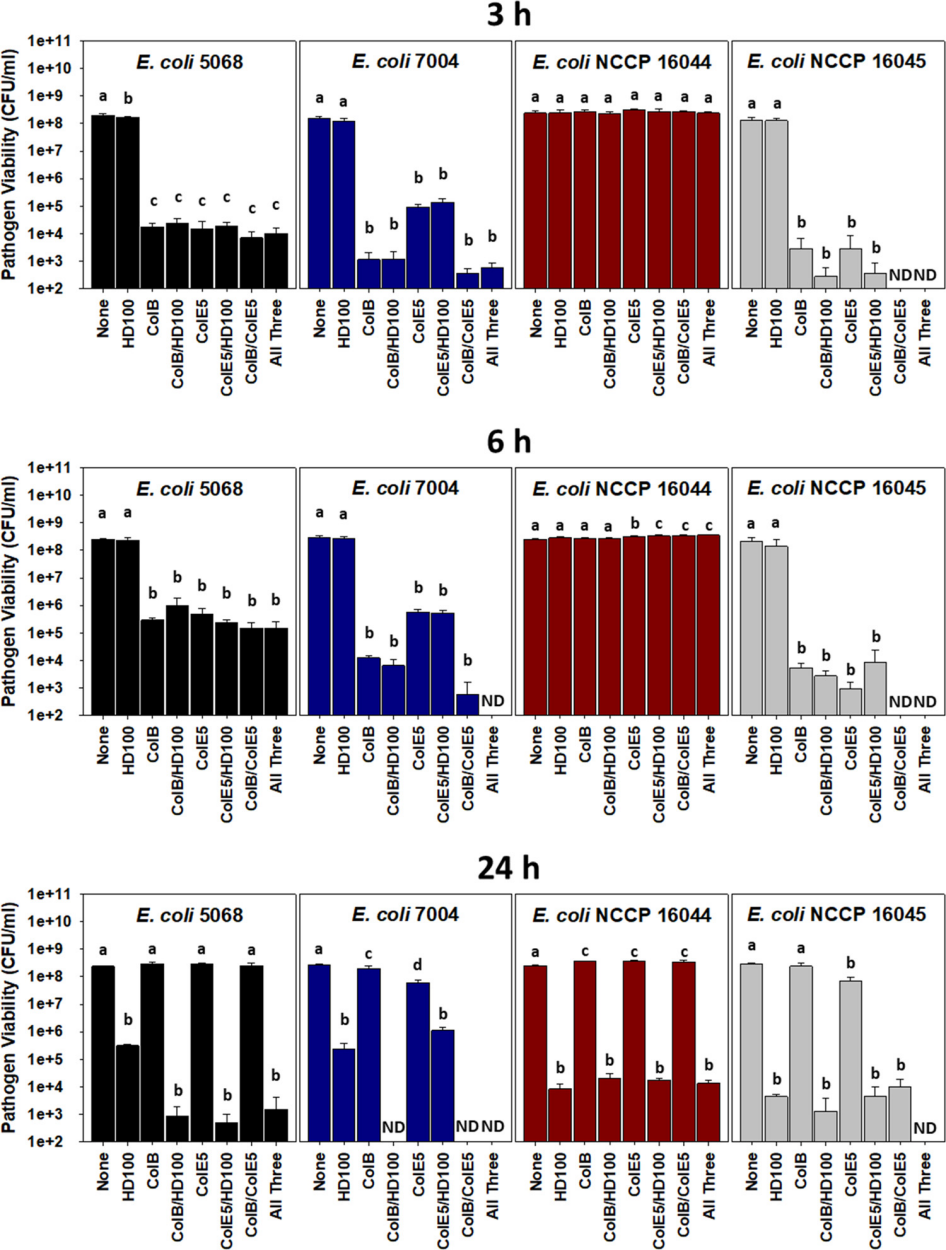
Cotreatment with B. bacteriovorus HD100 and the colicins enhances killing of colistin- and carbapenem-resistant pathogenic E. coli strains. The results presented are the E. coli viabilities in DNB medium at 3, 6 or 24 h for each of the treatments. The results show the colicins, when used individually and together/in combination, were active early on (3 and 6 h) but each of the susceptible pathogens grew thereafter. The predator was active later, causing significant drops in the pathogen viabilities at 24 h. When used together, i.e., both colicins or the colicins and predator, better killing efficiencies were seen throughout for several of the pathogenic E. coli strains. One-way ANOVA was performed followed by a Tukey *post hoc* test for each strain and time point. Statistically significantly groups (*P*-value < 0.05) are indicated on the graph using letters (a, b, c and d). ND – not detected (<100 CFU/mL). (*n *= 4).

Consequently, tests were performed once more with each E. coli pathogenic strain using the predator in conjunction with one or both colicins. As shown in Fig. 2, clear benefits were evident when individual colicins were used together with *B. bacteriovorous* HD100. At both 3 and 6 h, the colicins quickly killed the susceptible E. coli populations while the predator had no obvious effects. By 24 h, however, the *B. bacteriovorous* HD100 activities were evident, which improved the killing efficiencies in several of the cultures. This was the case for E. coli 5068 where the 24-h viabilities of this pathogen were 212- to 388-fold lower than predation alone when ColB or ColE5 were included, respectively. Similarly, the use of both ColB and the predator together completely eradicated E. coli 7004 cultures; i.e., their 24-h viabilities were less than 100 colony forming units (CFU) per mL. While this hinted at clear advantages when using *B. bacteriovorous* HD100 with individual colicins, the results with E. coli NCCP 16044 and E. coli NCCP 16045 found their combined activities were no better than when the predator was used alone. This was thought to be due to rapid development of resistance against the colicins mentioned above (Fig. S1).

A common method to prevent the development of resistance is the use of more than one colicin, as this reduces the chances for a resistant population to form (19, 20). Consequently, experiments were performed using ColB and ColE5 together. The results for each pathogen, however, were disparate (Fig. 2). For instance, E. coli 5068 was as sensitive to the ColB/ColE5 mixture as either colicin when they were used alone, while E. coli NCCP 16044 was once more completely resistant. In contrast, the ColB/ColE5 mixture was more potent than the individual colicins against both E. coli 7004 and E. coli NCCP 16045. This was particularly true for the E. coli NCCP 16045 cultures, which saw their viabilities drop precipitously below the detection limit (100 CFU/mL) at 3 h, even though some growth was observed at 24 h. The opposite was true for E. coli 7004 as its cultures were completely eradicated by the ColB/ColE5 mixture at 24 h (<100 CFU/mL; Fig. 2). For the same two pathogens (E. coli 7004 and E. coli NCCP 16045), addition of the predator improved the overall killing efficiencies and kinetics as the E. coli 7004 cultures were completely killed in 6 h (<100 CFU/mL) and remained so at 24 h, while no rebound growth was seen for E. coli NCCP 16045. The results with E. coli NCCP 16045 were also verified using confocal microscopy, where a dual treatment with the colicins effectively reduced the presence of this pathogen, but no E. coli cells were seen when a combined treatment of the colicins and predator was used (Fig. S4).

In conclusion, this study illustrates potential benefits when predatory strains and colicins are used together to control and combat pathogenic E. coli populations. Here, we demonstrated the complementary nature of these two antibacterials to minimize the limitations of each, namely, incomplete killing of the pathogen by the predator and the development of resistant populations with the colicins, as well as their combined potential to completely eradicate pathogenic E. coli populations. While focus within this study was given solely to colistin- and carbapenem-resistant clinical isolates of E. coli and the activities of only ColB and ColE5, diverse classes of bacteriocins are known to exist that target other priority pathogens (1), including Klebsiella pneumoniae (klebicins [21]), Salmonella spp. (salmocins [22]) and even Gram-positive pathogens, such as Staphylococcus spp. (23). In addition to being active against diverse pathogens, bacteriocins are quite diverse in their antibacterial mechanisms (1), including inhibition of peptidoglycan synthesis (24), RNase (25), and DNase (26) capabilities. This is particularly relevant as the predatory-colicin mixtures led to better killing efficiencies with each of the colicin-sensitive strains, even though some variability in activity was observed according to the pathogen. Extending this to the ColB- and ColE5-resistant E. coli NCCP 16044, if a colicin that is active against this pathogen is identified, it can potentially be applied in a cotreatment with B. bacteriovorus HD100 against this pathogen. Similarly, a combinatorial approach like that described previously (19), where five colicins were used at once to prevent resistance development and increase efficacy, can be used alongside B. bacteriovorus to kill pathogenic E. coli strains that may be naturally resistant to a given colicin. Given plant-produced colicins recently received a favorable regulatory review as being generally regarded as safe (GRAS) (27) and are generally effective at controlling pathogens on foodstuffs (27–29), this represents one possible avenue for their coapplication alongside BALOs to reduce or prevent foodborne outbreaks. Other possibilities include their combined use to treat abiotic surfaces, such as stainless steel tabletops, where bacterial pathogens may be present and form biofilms (30). As such, the results of this study present just a small glimpse of the possibilities available where bacteriocins are used alongside predatory bacteria to control drug-resistant pathogens, a marriage that demands further exploration and consideration.

### Ethical statement.

This article does not contain any studies with human participants or animals performed by any of the authors.
