# Supplementary material for: Pairing Colicins B and E5 with Bdellovibrio bacteriovorus To Eradicate Carbapenem- and Colistin-Resistant Strains of Escherichia coli
Source: Microbiol Spectr. 2023 Apr 10;11(3):e00173-23. doi: 10.1128/spectrum.00173-23 (PMC10269710; doi:10.1128/spectrum.00173-23)
Supplement: Supplemental file 1 — Supplemental material. Download spectrum.00173-23-s0001.pdf, PDF file, 1.0 MB [file spectrum.00173-23-s0001.pdf]

Supplemental Information and Data

for

Pairing Colicins B and E5 with *Bdellovibrio bacteriovorus* to Eradicate Carbapenem- and Colistin-Resistant Strains of *Escherichia coli*

Sumudu Upatissa[†], Wonsik Mun[*] and Robert J. Mitchell[*]

School of Biological Sciences

Ulsan National Institute of Science and Technology (UNIST)

Ulsan, South Korea.

Corresponding Authors

WM – wmun@unist.ac.kr

RJM – esgott@unist.ac.kr

Table S1. Bacterial strains used in this study

Bacterial Strain	Characteristics	Reference
E. coli 5068	Clinical isolate; Colistin resistant (*mcr-1*)	(1)
E. coli 7004	Clinical isolate; Colistin resistant (*mcr-1*)	
E. coli NCCP 16044	Clinical isolate; Carbapenem resistant (*bla*$_{KPC-2}$)	
E. coli NCCP 16045	Clinical isolate; Carbapenem resistant (*bla*$_{OXA-232}$)	
B. bacteriovorus HD100	Predatory bacterium	(2)

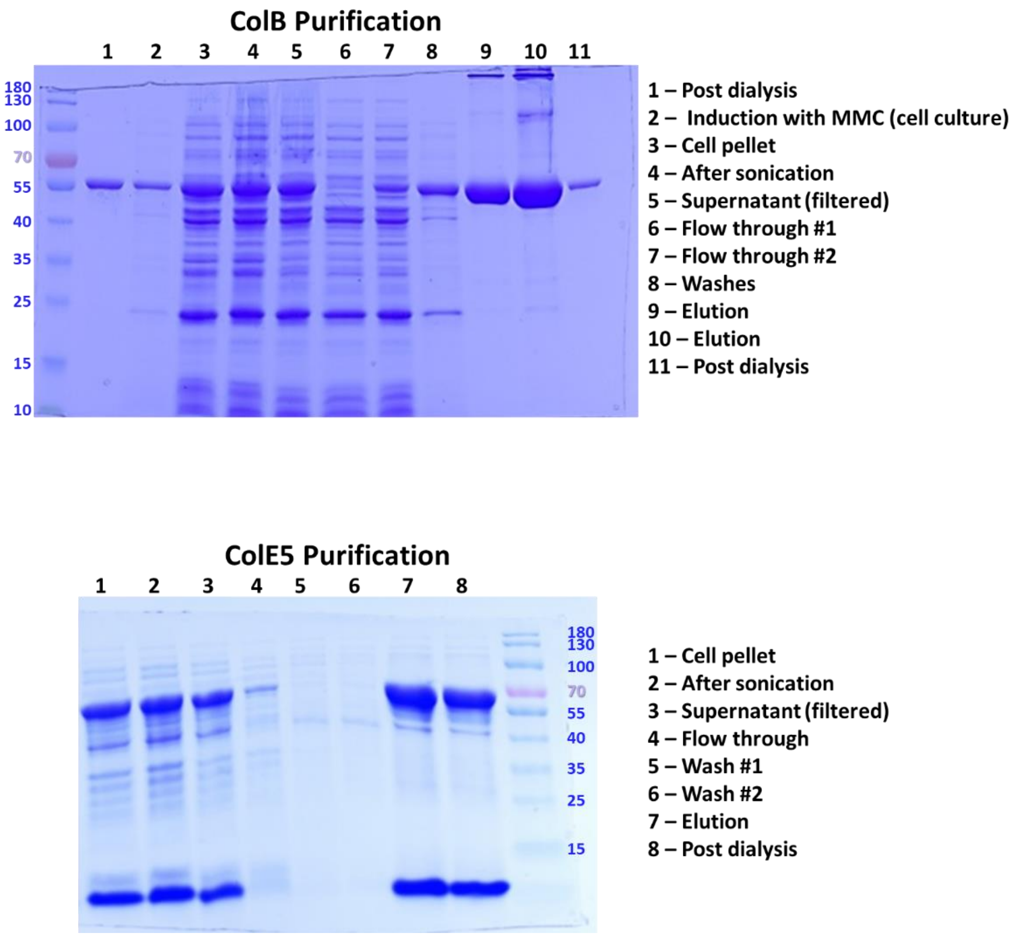

Figure S1. SDS-PAGE gel images showing the column affinity purification of both colicins. (Top) ColB purification. (Bottom) ColE5 purification. In the ColE5 gel, the immunity protein is clearly evident at the bottom of the image. Its absence in the ColB may be due to it being lost off the edge of the gel.

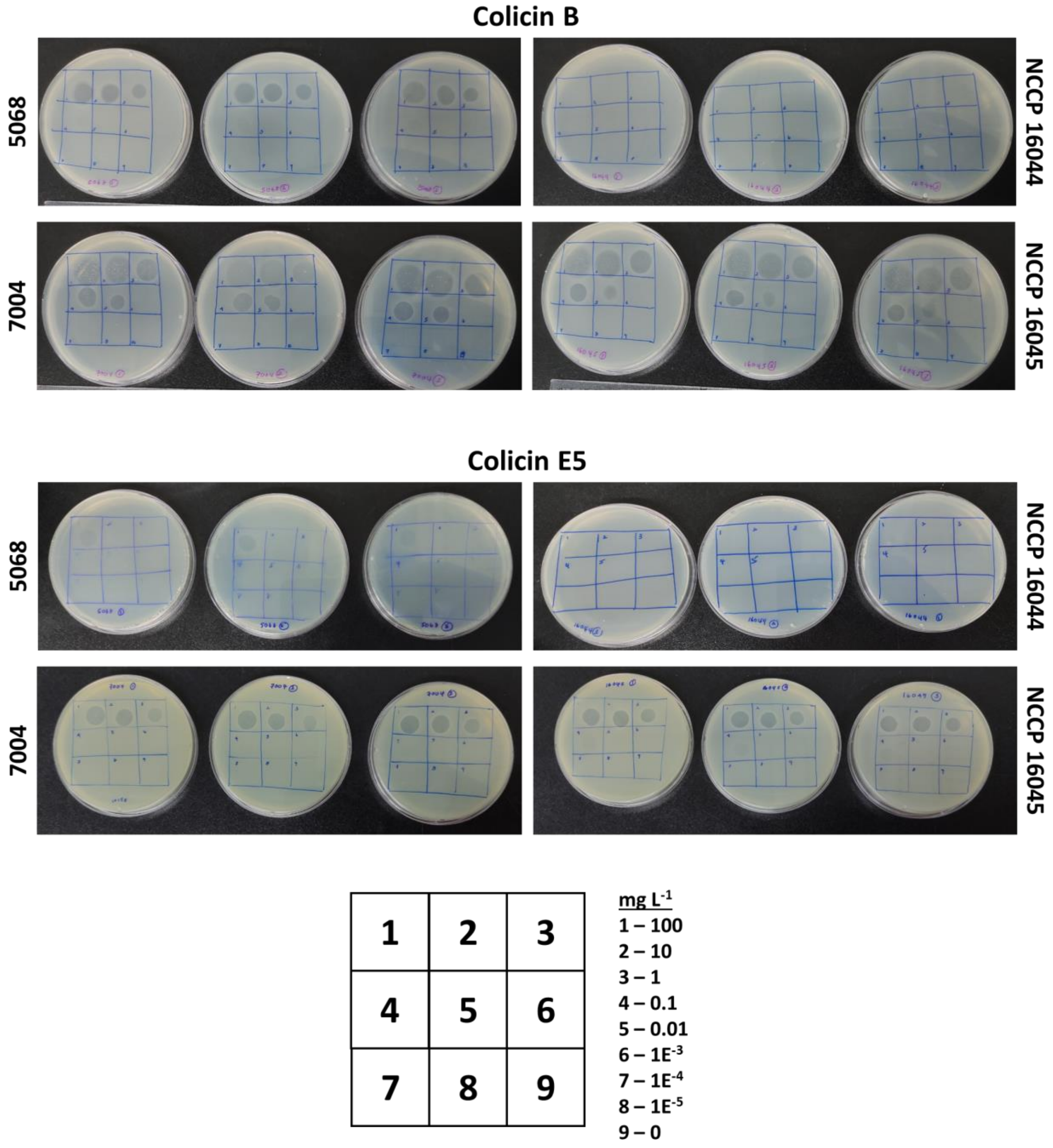

Figure S2. Spot titer plates to evaluate the activity and concentration needed for ColB and ColE5. Serial dilutions of the colicins were spotted onto tryptone top agar plates containing the different clinical *E. coli* strains. The results show *E. coli* strains 5068, 7004 and 16045 were all susceptible to both colicins, although 5068 was only slightly so with ColE5 at the highest concentration tested. Agreeing with Figure 1a, however, *E. coli* 16044 was resistant to both colicins. (n = 3)

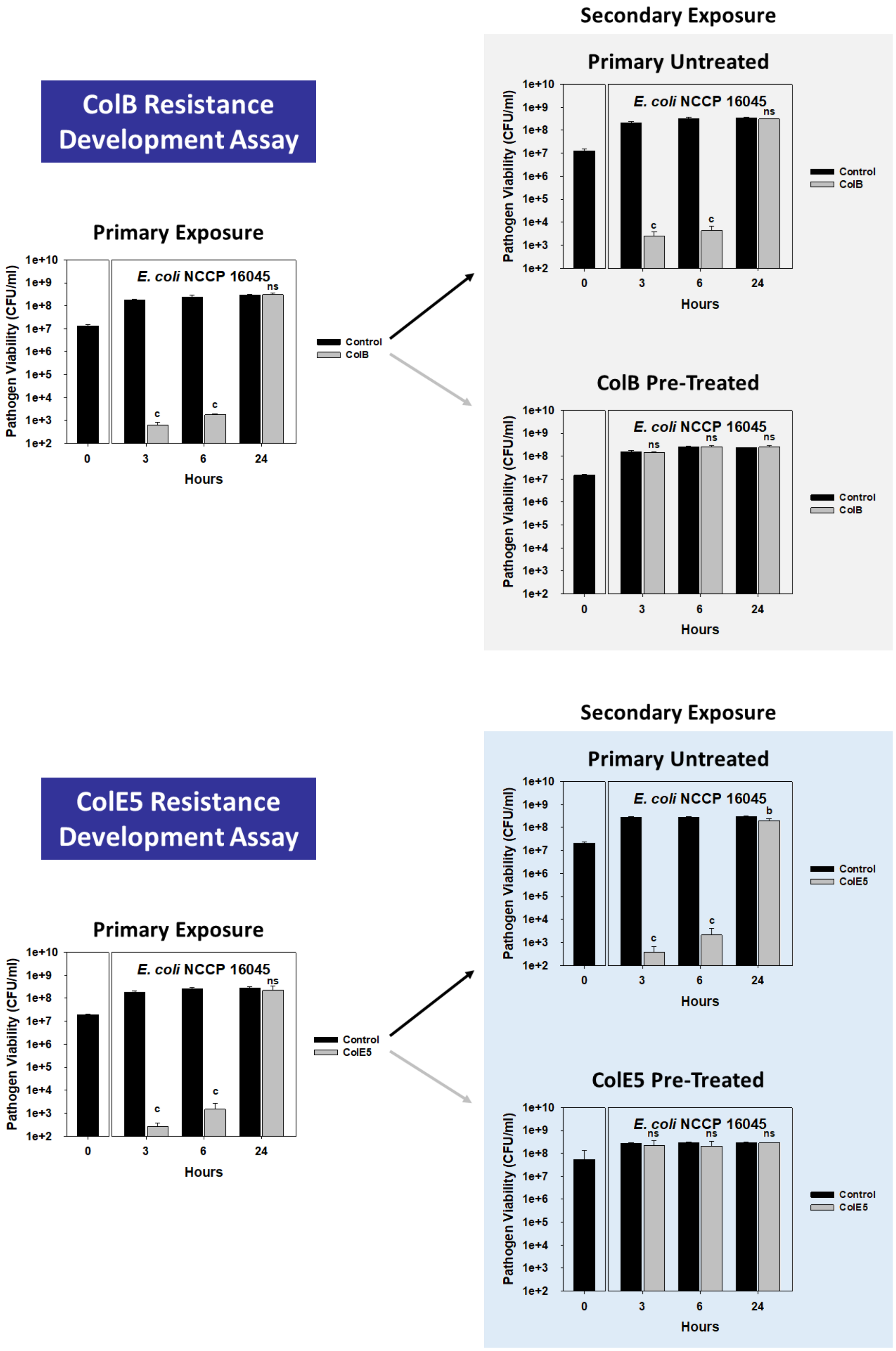

Figure S3. Pre-exposure to ColB or ColE5 leads to resistance development. Treatment of *E. coli* NCCP 16045 with 10 mg L^{-1} of either ColB or ColE5 caused a resistant phenotype to develop in the surviving populations. (Top) ColB. (Bottom) ColE5. $b - p < 0.01$; $c - p < 0.001$; $ns - p > 0.05$. ($n = 4$)

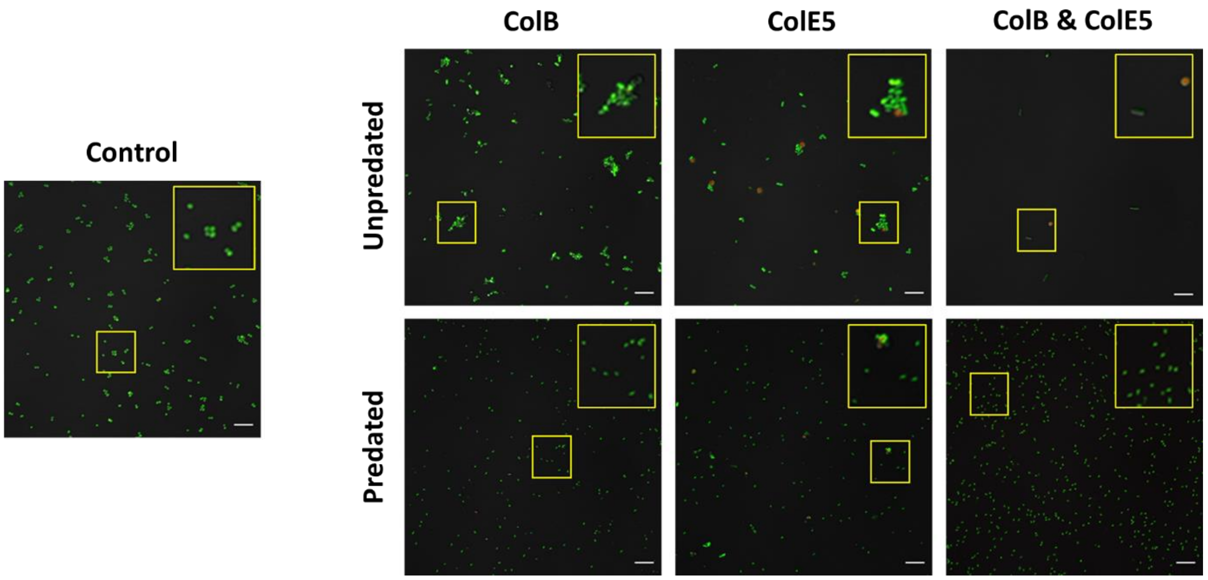

Figure S4. Live/Dead imaging of *E. coli* NCCP 16045 cultures after treatment for 24 hours. The results show similar numbers of live *E. coli* when either ColB or ColE5 were used, agreeing with the results in Figure 1a. When both colicins were used, however, the number of cells visually present dropped and very few cells were obviously viable. This supports the findings in Figure 2 where the combined activities of these two colicins caused a nearly 30,000-fold drop in the 24-h viabilities for this pathogen. Similarly, the predated samples where ColB or ColE5 were used alone with *B. bacteriovorus* HD100 had only a few *E. coli* present within them (the small green bacteria are the predator) while the culture treated with all three antibacterials found only the predator present.

Experimental

Bacterial strains and their growth

Bacterial strains are listed in Table S1. The pathogenic *E. coli* clinical isolates used in this study are *E. coli* 5068 (1) and *E. coli* 7004, both of which are colistin-resistant, and the carbapenem-resistant *E. coli* NCCP 16044 and *E. coli* NCCP 16045. Overnight cultures of these bacterial strains were prepared in lysogeny broth (LB) at 37ºC and 250 rpm. They were then sub-cultured in fresh LB medium (1:100 dilution) and grown to the mid-logarithmic stage (optical density at 600 nm (OD) of 0.5), after which they were pelleted (7000 x g for 5 min) and resuspended in dilute nutrient broth (DNB (0.1x NB)) supplemented with 3 mM $MgCl_2$ and 2 mM $CaCl_2$ to an OD of 0.1. The predatory strain used in this study is *Bdellovibrio bacteriovorus* HD100. This strain was routinely grown using *E. coli* MG1655, a non-pathogenic strain, as the prey host as described previously (2, 3) in DNB medium supplemented with 3 mM $MgCl_2$ and 2 mM $CaCl_2$. Briefly, after growth of *E. coli* MG1655 overnight in LB medium, the prey cells were pelleted (7000 x g, 5 minutes) and resuspended to an optical density (OD; 600 nm) of 1.0 in sterile DNB buffer with 3 mM $MgCl_2$ and 2 mM $CaCl_2$ salts added (4). To these cultures, the predator was added (1:100 (v:v) dilution of the 0.45 µm-filtered predatory culture), corresponding to an initial predator-to-prey ratio (PPR) of approximately 0.03. These cultures were then grown at 30 ºC and 250 rpm overnight. After predation was complete, the culture was filtered (0.45 µm syringe filter, Millipore, USA) to purify the predator away from any remaining prey cells or bdelloplasts.

Production and purification of the colicins

The colicins (ColB and ColE5) and their immunity proteins were expressed from plasmids pKP864 (Addgene plasmid # 167658; http://n2t.net/addgene:167658; RRID:Addgene 167658),

and pET21::colE5-immE5 (5, 6), respectively, in *E. coli* BL21(DE3) and purified using the protocols described previously (5-7). Briefly, to expresses the colicins, cultures of *E. coli* BL21(DE3) were grown overnight in LB medium (30 ºC and 250 rpm) with either 30 mg/L chloramphenicol (pKP864) or 100 mg/L ampicillin (pET21::colE5-immE5), and then inoculated (1:100 dilution) into fresh LB (100 ml). When the ODs reached 0.5, either mitomycin C (MMC; 1 mg/L final concentration) or IPTG (1 mM final concentration) were added to induce production of ColB or ColE5, respectively. After incubating for another six hours, the cells were pelleted (7000 x g, 5 min) and stored at -80℃ until protein purification.

Both of these proteins were expressed with a His-tag epitope attached directly to the colicin or its immunity protein, *i.e.*, to ColB in pKP864 and to the ImmE5 immunity protein in pET21::colE5-immE5, which was used to purify the colicin. For this, the bacterial pellets were resuspended in 5 ml of either 50 mM Tris-HCl (pH 7.5) (ColB) or 20 mM sodium phosphate (pH 7.0) containing 150 mM NaCl (ColE5). In both cases, 1 mM phenylmethylsulfonyl fluoride (PMSF) was added as a protease inhibitor. After sonication, the samples were centrifuged (16000 x g, 30 min) to clarify the samples by removing intact cells and cell debris. Purification of the colicins was then performed using Ni^{2+}-NTA columns (HisPur Ni-NTA Spin Columns, Thermo-Fisher Scientific (USA)) according to the manufacturer's suggested protocol. Briefly, the clarified cell lysate was loaded onto the Ni^{2+}-NTA column and allowed to equilibrate with shaking for 30 min at 4℃. The column was then centrifuged (700 x g, 2 min) and the flow-through collected. The column was washed three times with wash buffer (10 mM imidazole in either 50 mM Tris-HCl or 20 mM sodium phosphate (pH 7.0)/150 mM NaCl) before the colicin was eluted using 250 mM imidazole in the respective buffer. The flow-through was collected at each stage of washing and elution to evaluate the purity of each sample via 10 % SDS-PAGE (Figure S2). After pooling positive samples, they were dialyzed overnight at 4ºC, with gentle stirring, against the respective buffer for each colicin using a 15-kDa

molecular weight cut-off tubing (Tube-O-DIALYZER mini dialysis system, Merck). The concentration of each purified colicin was measured using the Bradford assay before being mixed with sterile glycerol (final concentration – 10%) and stored as aliquots at -80ºC. As needed, aliquots of the colicin stocks were thawed and diluted into their respective buffers before use. Samples (50 µl) from each step of the purification were kept for the protein gel analyses (Figure S3).

Spot titer assay

Each of the four *E. coli* clinical isolates was grown overnight in LB medium as described above before being diluted (1:100) into fresh LB. When the OD was 0.5, 100 µl of the cell culture was mixed with 3 ml of molten (45ºC) tryptone top agar (10 g-L^{-1} tryptone, 5 g-L^{-1} NaCl, 7 g-L^{-1} agar) and layered on tryptone agar (10 g-L^{-1} tryptone, 5 g-L^{-1} NaCl, 15 g-L^{-1} agar) plates. After solidifying, 10 µl of the diluted colicin solutions were spotted on the plate. The plates were then incubated overnight at 30 ºC and the presence of a clear zone indicated the clinical isolate was susceptible (Figure S3).

Antibacterial activities of the colicins

Based on the spot titer assays, two doses were selected for further evaluation – 1 and 10 mg L^{-1}. The impacts of these doses on the viabilities of the four clinical isolates were performed in liquid cultures. Briefly, the cultures were grown as above at 30 ºC until the OD reached 0.5, at which point the cells were pelleted (7000 x g, 5 min) and resuspended to an OD of 0.1 in DNB medium supplemented with 3 mM $MgCl_2$ and 2 m$MCaCl_2$. Aliquots (180 µl) were added to the wells of a 96-well plate (white, Greiner) and 20 µl of the diluted colicin stocks were added. Control wells had only 20 µl of DNB added (*i.e.*, no colicin). The plate was then incubated at 30 ºC and 250 rpm for 24 hours, during which the viabilities of the clinical isolates were

determined by serial dilution and plating on LB agar plates. These plates were incubated at 37 ºC overnight for the colonies to grow and develop. The impacts of the colicins on *B. bacteriovorus* HD100 viabilities were performed using a similar protocol. The *E. coli* and predatory viabilities under each test condition were evaluated using the resazurin assay described previously (3) in parallel with viable counts. The data presented is the colony-forming units (CFU) and plaque-forming units (PFU) for the prey and *B. bacteriovorus* HD100, respectively.

Antibacterial activities of *B. bacteriovorus* HD100

These experiments were performed as described previously (8), albeit with DNB and an initial predator-to-prey ratio (PPR) of approximately 0.25. Briefly, the predator was grown using *E. coli* MG1655 as the prey as described above and then filtered (0.45 µm, Millipore). The predatory culture was then diluted (1:100) in DNB and mixed equally (100 µl each) with the prepared prey cultures (OD 0.2) in the wells of a 96-well plate (white, Greiner), giving an initial PPR that was consistently between 0.2 and 0.3. Control wells had the prey mixed with only sterile DNB medium (*i.e.*, no predator). These cultures were grown for 24 hours at 30 ºC and 250 rpm, during which time the viabilities of the prey were measured using serial dilutions and plating as described above.

Combined activities of the colicins and *B. bacteriovorus* HD100

The combined activities of both antibacterials were performed using basically the same protocols as described above for the predator except 20 µl of the diluted colicin stock was also added to the wells. The control wells had the prey mixed with only sterile DNB medium (*i.e.*, no predator) and 20 µl of the respective buffer without the colicin.

Live/dead microscopic imaging

Microscopy samples of the bacteria were collected after a 24-h exposure of *E. coli* NCCP 16045 to either the colicins alone or with the predatory bacteria as described above. Live/Dead staining was performed using LIVE/DEAD™ BacLight™ (Invitrogen) following the manufacturer's protocol. Briefly, each of the stock solutions was added as a working concentration to 1 ml of the sample. The sample was incubated at RT for 20 minutes, pelleted (7000 x g, 5 minutes) and 950 µl of supernatant was carefully aspirated off. The pellet was then resuspended by pipetting in the remaining 50 µl, and 1 µl of this sample was transferred to a coverslip to observe the bacteria within using confocal microscopy (Carl Zeiss, LSM780). The fluorescence from the stained samples were detected using excitation/emission wavelengths of 480nm/500nm (Live) and 490nm/635nm (Dead).

Reproducibility and statistical analysis

Unless specified, each experiment was performed in quadruplicate for each data set, plotted as the averages and the standard deviations presented on the graphs as error bars. Normal distribution of each dataset was verified using the Shapiro-Wilk test. For those samples that did not show a substantial departure from the normality ($p > 0.05$), the student T-test was used. When multiple data sets were compared against each other, the analysis of variance (ANOVA) test was performed followed by Tukey post-hoc analyses. Groups that are statistically significant at p-value < 0.05 are shown in the graph using letters (a, b, c, and d).

References

1. Jang H, Choi SY, Mun W, Jeong SH, Mitchell RJ. 2022. Predation of colistin- and carbapenem-resistant bacterial pathogenic populations and their antibiotic resistance genes in simulated microgravity. Microbiological Research 255.
2. Mun W, Upatissa S, Lim S, Dwidar M, Mitchell RJ, Kim M. 2022. Outer Membrane Porin F in E. coli Is Critical for Effective Predation by Bdellovibrio. Microbiology Spectrum 10.
3. Jang H, Mun W, Choi SY, Mitchell RJ, Khursigara CM. 2022. Use of Resazurin To Rapidly Enumerate Bdellovibrio and Like Organisms and Evaluate Their Activities. Microbiology Spectrum 10.
4. Mun W, Kwon H, Im H, Choi SY, Monnappa AK, Mitchell RJ. 2017. Cyanide Production by Chromobacterium piscinae Shields It from Bdellovibrio bacteriovorus HD100 Predation. mBio 8.
5. Beck CM, Willett JLE, Cunningham DA, Kim JJ, Low DA, Hayes CS. 2016. CdiA Effectors from Uropathogenic Escherichia coli Use Heterotrimeric Osmoporins as Receptors to Recognize Target Bacteria. PLOS Pathogens 12.
6. Beck CM, Diner EJ, Kim JJ, Low DA, Hayes CS. 2014. The F pilus mediates a novel pathway of CDI toxin import. Molecular Microbiology 93:276-290.
7. Hilsenbeck JL, Park, H., Chen, G., Youn, B., Postle, K., & Kang, C. 2004. Crystal structure of the cytotoxic bacterial protein colicin B at 2.5 Å resolution. Molecular microbiology 51:711-720.
8. Dwidar M, Jang H, Sangwan N, Mun W, Im H, Yoon S, Choi S, Nam D, Mitchell RJ. 2020. Diffusible Signaling Factor, a Quorum-Sensing Molecule, Interferes with and Is Toxic Towards Bdellovibrio bacteriovorus 109J. Microbial Ecology 81:347-356.
